# Comorbidity Genes of Alzheimer’s Disease and Type 2 Diabetes Associated with Memory and Cognitive Function

**DOI:** 10.3390/ijms25042211

**Published:** 2024-02-12

**Authors:** Seong Beom Cho

**Affiliations:** Department of Biomedical Informatics, College of Medicine, Gachon University, 38-13, Dokgeom-ro 3 Street, Namdon-gu, Incheon 21565, Republic of Korea; sbcho1749@gachon.ac.kr

**Keywords:** Alzheimer’s disease, type 2 diabetes, comorbidity, memory, cognition

## Abstract

Alzheimer’s disease (AD) and type 2 diabetes mellitus (T2DM) are comorbidities that result from the sharing of common genes. The molecular background of comorbidities can provide clues for the development of treatment and management strategies. Here, the common genes involved in the development of the two diseases and in memory and cognitive function are reviewed. Network clustering based on protein–protein interaction network identified tightly connected gene clusters that have an impact on memory and cognition among the comorbidity genes of AD and T2DM. Genes with functional implications were intensively reviewed and relevant evidence summarized. Gene information will be useful in the discovery of biomarkers and the identification of tentative therapeutic targets for AD and T2DM.

## 1. Introduction

Alzheimer’s disease (AD) is a neurodegenerative disorder characterized by memory and cognitive impairment. The main pathology of AD is the accumulation of beta-amyloid (Aβ), which is believed to cause the main symptoms of the disease [1]. Clearing Aβ or tau proteins that are alleged to induce AD has been the main strategy in the development of therapeutic agents; however, the results of clinical studies have been unsatisfactory, and there has been no definite treatment discovered for AD [2]. This may be due to the fact that the accumulation of Aβ and tau proteins has not always been correlated with clinical outcomes.

It is well known that type 2 diabetes (T2DM) co-occurs with AD [3]. There is significant evidence that the two diseases develop concomitantly, and the comorbidity relationship is based on the shared molecular mechanisms between AD and T2DM [4]. Moreover, genes involved in comorbidity can be a valuable resource for drug repurposing [4,5]. Therefore, it is desirable that the identification of comorbidity genes for AD and T2DM provides clues for further development of AD drugs or management strategies.

In this review, disease genes gathered from previous studies were used and protein–protein interaction network-based clustering (Markov clustering algorithm) was applied for the identification of comorbidity genes of AD and T2DM that are related to memory and cognitive function. For this purpose, genes involved in memory and cognitive functions were also collected, and the intersections of these genes and the comorbidity genes were applied to the clustering.

## 2. Biological Mechanisms of Alzheimer’s Disease and Type 2 Diabetes 

Further, in the enrichment analysis of 7763 gene ontology (GO) biological processes (GOBPs), 2857 statistically significant terms were identified (Table 1 and Table S2), with “RESPONSE_TO_OXYGEN_CONTAINING_COMPOUND” being the most significant one (odds ratio = 16.06, *p* = 1.97 × 10^−301^). Additionally, “REGULATION_OF_CELL_DEATH” was among the most highly ranked GOBPs (odds ratio = 11.97, *p* = 5.53 × 10^−222^).

ORA with KEGG (Kyoto Encyclopedia of Genes and Genomes) pathway analysis revealed that 77 of the 173 enriched KEGG pathways were significant (Table S3). Signaling-related pathways ranked at the top (Table 2). “PATHWAYS_IN_CANCER” was the most significant pathway in the results and included several different pathways.

## 3. Gene Clusters of Common Genes That Are Associated with AD, T2DM, and Memory Function

Among the 1381 common genes of AD and T2DM, 361 genes overlapped with the memory-associated genes in the DisGeNet database (Table S1). Using the information on protein interactions in the STRING database, Markov clustering of the interaction network was performed using the default parameters, and 93 clusters with different numbers of genes ranging from 1 to 13 were obtained. Table S4 lists these clusters and their associated proteins. The cluster numbers were determined according to the average local clustering coefficient of the network-based clustering method. Therefore, the first cluster (Cluster 1) had the highest average local clustering coefficient, indicating tighter connections between the proteins within the cluster compared to the other clusters.

### 3.1. Cluster 1 (CL1)

**Figure 1:**
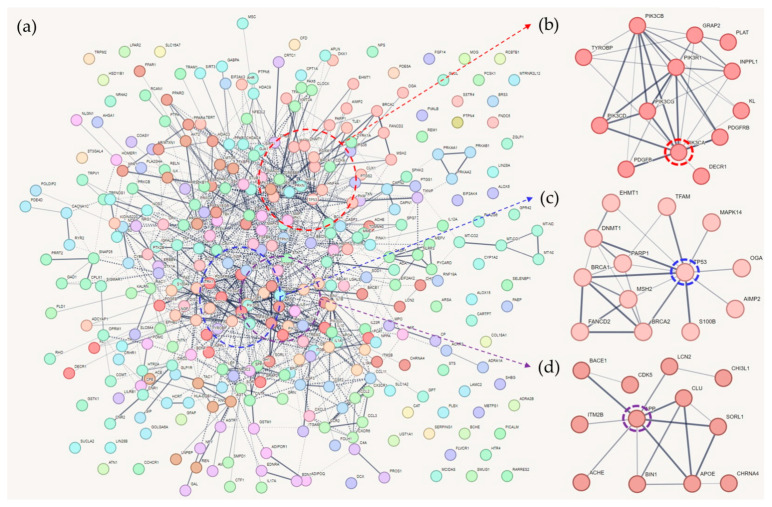
The result of network clustering with common genes of Alzheimer’s disease (AD), type 2 diabetes, and memory-associated genes: (**a**) total result; (**b**) in Cluster 1, PIK3C genes constitute hub proteins of the cluster; (**c**) TP53 is the hub protein of Cluster 2; (**d**) APP, well known for its roles in AD, is the hub gene of Cluster 3. Nodes with the same colors indicate the same clusters.

CL1 included 13 genes (Figure 1). PI3K and PDGF-related genes were frequent in this cluster.

PI3K is a well-known enzyme involved in various cellular functions, including apoptosis, glucose uptake, and neuroprotection [6]. Many PI3K family members (PI3K subtypes) function in the Akt and mTOR pathway [6,7]. In AD, the PI3K pathway is inhibited by Aβ, which has been linked to increased apoptosis of neurons [8]. Moreover, the PI3K/Akt signaling pathway is involved in tau phosphorylation, dysregulated insulin signaling, suppression of autophagy through the activation of mTOR, and altered responses to oxidative stress in patients with AD [8,9]. PI3K plays a role in glucose uptake by muscle and adipose cells [10], and abnormal PI3K signaling causes insulin resistance in animal models [11]. The PI3K-related pathways, including Akt and mTOR, are associated with neuronal development and brain memory function [12,13,14]. PI3K subtypes PIK3CA, PIK3CB, PIK3CD, PIK3CG, and PIK3R1 were all included in CL1. 

*PIK3CA* was a hub gene in CL1; it is predicted to be involved in the immune-related phenomena of AD development [15]. In an AD zebrafish model, 20S-protopanaxatriol (PPT) facilitated neurogenesis of neural stem cells (NSCs) and reduced NSC apoptosis and cell cycle arrest by Aβ (which might hinder PIK3CA and PPT binding) [16]. Bioinformatics analysis of molecular docking and identification of network modules revealed that PIK3CA was one of the target genes for Byu dMar 25 (BM25), a molecule known to have therapeutic potential in AD [17]. When frog skin peptide, which is a stimulant of insulin release, was administered to a T2DM mouse model, the expression of Pik3ca (the mouse ortholog) increased in skeletal muscles [18,19]. PIK3CB has been associated with insulin resistance and hepatic glucose production according to promoter variants [20,21,22]. The expression of PIK3CB is downregulated in patients with AD and linked to the apoptosis and axon guidance pathways [23]. PIK3CB is also genetically associated with mild cognitive impairment (MCI) showing abnormalities in temporal lesions that modulate memory function [24]. *PIK3CD* mRNA in peripheral leukocytes is upregulated in patients with gestational diabetes, whereas in patients with T2DM treated with sitagliptin it is downregulated [25,26]. Similar to PIK3CB, PIK3CD is also genetically associated with MCI [24]. PIK3R1 is well known for its relationship with T2DM and insulin resistance [27,28]. Mutations in *PIK3R1* cause SHORT (short stature, hyperextensibility of joints and/or inguinal hernia, ocular depression, Rieger anomaly, and teething delay) syndrome and accompanying T2DM [29,30,31]. Moreover, the analysis of exome sequencing data from over 10,000 subjects in the Alzheimer’s Disease Sequencing Project showed evidence of a functional variant of *PIK3R1* [32]. Coexpression network analysis has revealed that *PIK3R1* is one of the core immune genes involved in AD and that it is associated with Aβ and tau protein pathology [33]. 

CL1 included two PDGF-related proteins, PDGFB and PDGFRB. PDGF is associated with vascular complications in T2DM [34] and cell death caused by Alzheimer-associated neuronal thread protein [35]. PDGFB and PDGFRB are also involved in vascular complications of T2DM [36,37]. In AD, PDGFRB activation has a mitogenic effect that is blocked by Aβ, preventing the neuroprotective effects of PDGF-BB [38]. Mutations in these two genes cause brain calcifications [39,40], which can be observed in patients with AD [41]. 

### 3.2. Cluster 2 (CL2)

In CL2, *P53* acted as a hub gene by showing the strongest connectivity (Figure 1). P53 has a neuroprotective effect by repressing BACE1 and thus the Aβ production cascade. Interestingly, Aβ may also repress *P53* expression in AD [42]. Moreover, MCI is affected by conformational changes in P53 [16,43]. It is well known that cancer and AD have an inverse correlation in incidence, and the underlying molecular mechanisms seem to involve *P53* and related genes [44,45]. Phosphorylated forms, genetic variations, and unfolded P53 have been proposed as biomarkers for AD [44,46,47]. P53-related novel mechanisms, including mitochondrial dysfunction and overexpression of CDK5 in AD and other neurodegenerative diseases, have also been proposed as biomarkers [46,48]. In previous studies, genetic variants of *P53* have also been associated with T2DM [49,50,51]. Therefore, *P53* has been identified as one of the hub genes involved in the pathogenesis of AD and T2DM [52]. Notably, P53 also regulates pancreatic cell survival and glucose homeostasis [53]. 

BRCA1 plays a role in repairing DNAs under stress, including the stresses caused by ultraviolet light and reactive oxygen species, and failures of this mechanism in neurons may be related to AD [54,55]. Downregulation of *BRCA1* and other DNA repair genes has been observed in patients with clinically evident AD [56]. *BRCA1* depletion was shown to impair cognitive function in mice [57]. In addition, abnormal accumulation of P53 occurs in AD and other tauopathies [58,59], and may be caused by hypomethylation of the promoter region of *P53* [60]. BRCA1 is known to interact with acetyl coenzyme A (CoA) carboxylase α (ACCA), which results in lipogenesis [61]. Hypermethylation of BRCA1 was observed in patients with T2DM [62]. 

S100B is well known for its role in AD. S100B is involved in gliosis and inflammatory reactions and suppresses the neurodegeneration of cholinergic neurons in mouse models of AD [63,64]. Additionally, S100B is associated with memory and cognition. The inhibition of IL-1, for example, decreases levels of S100B, leading to an alleviation of cognitive deficits and tau production [65]. Neutralization of S100B in a rat sepsis model increased cognitive performance scores [66], and pharmaceutical suppression of S100B reduced gliosis and neuronal loss [67]. Furthermore, it has been shown that S100B and the receptor for advanced glycation products (RAGE) affect learning and memory impairment by interacting with IL-1, IL-6, and TNF-α [68]. Serum S100B levels were positively correlated with cognitive performance tests in a healthy elderly population [69]. In contrast, they also showed a positive correlation with AD severity [70]. S100B is also associated with the pathophysiology of T2DM. In a mouse model, S100B induced beta cell apoptosis [71]. Serum S100B levels were elevated in patients with T2DM with peripheral neuropathy [72], and S100B levels correlated with cognitive performance in patients with T2DM [73]. In the coronary arterioles of a mouse model, S100B suppressed the vasodilatation effect of acetylcholine [74]. 

DNMT1 is an enzyme that catalyzes the transfer of methyl groups to DNA CpG sites, and previous research in animal models has shown that aberrant DNMT1 expression is associated with memory impairment [75,76,77,78]. In a high methionine-induced AD rat model, *DNMT1* was downregulated and tyrosine receptor kinase-induced memory impairment was observed [79,80]. In humans, DNMT1 has been associated with both AD [81,82,83,84], and T2DM, and increased *DNMT1* expression has been observed in beta islet cells from patients with T2DM [85]. IL-6, which is a major inflammatory mediator, induces insulin resistance and reduces DNMT1 protein levels in endothelial cells [86]. In CL2, poly(ADP-ribose) polymerase 1 (PARP1) was not directly connected to P53 but linked to it via DNMT1. 

In diabetic mice, NF-kB inhibition improves vascular function and increases cleaved PARP1 [87,88]. The role of PARP1 in T2DM was discovered through the modulation of PARP1 by diverse inhibitors. PARP1 inhibition reduces cardiac ischemia and inflammation in diabetic rats [89] and prolongs the lifespan of *Caenorhabditis elegans* under hyperglycemic conditions, probably via TCF7L2 [90]. PARP1 is associated with the vascular complications of T2DM, and has treatment potential for this condition [91,92,93,94]. Angiotensin II-treated heart muscles of diabetic mice showed elevated PARP1 activity, cardiac hypertrophy, and inflammation, conditions which were reversed by PARP1 inhibition [91]. Mendelian randomization identified a causal relationship between genetic variants of *PARP1* and obstructive coronary arterial disease in patients with T2DM [92]. When bromocriptine is used for the treatment of prolactinomas, it controls glucose and lipid profiles in diabetic rats, leading to changes in *p*-AKT followed by changes in Nf1 and PARP1 [93]. Cholesterol-induced lipotoxicity, which is related to beta cell dysfunction in obese patients with T2DM, has been shown to be controlled by the inhibition of PARP1 by GLP-1 administration [94]. 

### 3.3. Cluster 3 (CL3)

CL3 contained well-known AD-associated genes whose relatedness to T2DM has been less reported (Figure 1). Amyloid precursor protein (APP) is probably the most frequently studied molecule in AD research. Therefore, only APP studies related to memory or cognitive impairment were included in this review. For this purpose, a PubMed search was performed using “APP gene and Alzheimer’s disease and brain memory” as the keywords; the results included many studies on APP and their impact on memory function. JNK inhibition, for example, was shown to eliminate memory impairment and long-term potentiation deficits in a mouse model of AD in which APP phosphorylation was inhibited [95]. CRTC1 is a CREB coactivator whose expression is suppressed by APP [96]. When all-*trans* retinoic acid was administered to APP/PS1 transgenic mice, improved spatial learning and memory were observed compared to the control group, together with downregulation of CDK5 (a major kinase for APP and tau phosphorylation) [97]. According to a mouse model, low-density lipoprotein receptor-related protein 6 (LRP6) is involved in memory deficits via Wnt signaling, and the downregulation of this process is linked to the phosphorylation of APP and increased production of Aβ [98]. Additionally, APP haploinsufficiency prevented memory deficits in familial British dementia mouse models [99], and PTEN-induced putative kinase 1 (PINK1) was associated with memory impairment induced by APP [100]. Moreover, increased APP intracellular domain (AICD) production in hippocampal neurons has been shown to disrupt spatial memory [101]. Meanwhile, the role of APP in T2DM pathophysiology remains unclear, given that there is limited molecular evidence. However, it has been suggested that APP is the main regulator of insulin secretion in pancreatic islets [102]. Moreover, BACE2 (β-site APP-cleaving enzyme 2), a protease that is related to AD, is associated with insulin secretion in pancreatic islet cells [103]. BACE2 (β-site APP-cleaving enzyme 2) is a protease that is expressed in the brain and pancreas. In a mouse model overexpressing Islet Amyloid Polypeptide (IAPP), impaired glucose tolerance was observed. However, crossing this model with BACE2-deficient mice resulted in a significant improvement in glucose tolerance [104]. Imbalances in the production or removal of IAPP can lead to the rapid formation of cytotoxic amyloid fibrils. BACE2 is involved in processing IAPP in both the pancreas and the brain [105]. It has been reported that the overexpression of BACE2 increases the production of reactive oxygen species and decreases glucose-stimulated insulin secretion (GSIS), while the repression of BACE2 reverses this condition [103]. The therapeutic potential of BACE2 is currently under investigation [104,106,107].

Human APOE is a glycoprotein that is composed of 299 amino acids [108]. It is expressed in astrocytes and microglia and forms lipoprotein particles with cholesterol and its transporter [109]. APOE has a main role in redistributing cholesterol and other lipids [110]. APOE is a well-known AD biomarker. Moreover, the functional relationship between APOE and memory has been reported in many studies. When a proteomic analysis was applied to an AD mouse model, APOE was found to be differentially expressed in the hippocampus, which is related to memory function [111]. APOE is a transcriptional regulator of APP [112,113,114], and is involved in various biological pathways, such as the PGC-1alpha/sirtuin 3 axis, which alters mitochondrial function and, eventually, memory performance [115]. Multi-omics data analysis has revealed APOE haplotype-specific molecular alterations at both gene and protein expression levels [116]. The *APOE4* genotype induces an increase in unsaturated fatty acids and the accumulation of lipid droplets [117], and single-cell sequencing of postmortem human samples identified that some signaling pathways of cholesterol metabolism were altered in APOE4 carriers, resulting in reduced myelination [118]. The effects of APOE on brain function were confirmed using clinical data and imaging analyses. Using functional MRI analyses, APOE4 carriers performing moderate or severe working memory tasks showed less brain activation than non-APOE4 carriers [119]; APOE4 carriers also showed worse CA1 apical neuropil atrophy and episodic memory function [120]. APOE genotypes were found to be related to lower memory testing scores in patients with amnestic MCI and AD [121], lower memory performance in the normal elderly population [122], and reduced white matter connectivity [123], and gray matter volume [124]. APOE is associated with cardiovascular complications in patients with T2DM [125,126]. In particular, atherosclerosis and nephropathy are the most frequently reported complications associated with APOE genotypes [127,128,129,130,131,132]. As is well known, the APOE gene is a strong biomarker for Alzheimer’s disease (AD). APOE4 significantly increases the risk of AD, while APOE2 has a protective effect compared to APOE3 [133]. This indicates differential effects and multiple pathways for amyloid pathology. APOE is reported to be associated with tau pathology and decreasing efficiency in lipid transport, synaptic integrity and plasticity, glucose metabolism, and cerebrovascular function [134,135]. Recently, the risk associated with APOE4 has been known to be stratified according to ethnic groups, with the Hispanic population exhibiting the lowest level of risk associated with APOE4 [136]. Mechanistically, APOE has been associated with insulin resistance in the muscles of mouse models [137], islet amyloidosis [138], and adipocyte enlargement in atherosclerosis [139].

Clusterin (CLU) is a core protein in CL3; it is concurrently linked to APP and APOE. Studies of *CLU* gene variants and plasma protein levels have consistently revealed that CLU is associated with AD [140,141,142,143,144,145,146,147]. Molecular biology studies have identified the role of CLU in the pathophysiology of AD. In a CLU knockout mouse model, amyloid plaques were sparse in the cerebral parenchyma but prevalent in cerebral vessels, indicating that Aβ clearance had shifted to perivascular drainage [148]. CLU affects the lysosome pathway and Aβ processing in stem cell-derived neurons [149]. Additionally, overexpression of CLU in astrocytes ameliorates amyloid accumulation and gliosis [150]. It has also been found that the C allele of CLU is expressed at higher levels than other allelic variants and that C allele expression leads to exacerbation of inflammation and to an eventual inhibition of oligodendrocyte progenitor cell proliferation and myelination [151]. CLU is also associated with memory function. In a young population, working memory performance differed between CLU genotypes [152], and methylation around SNPs rs9331888 and rs9331896 in the *CLU* gene was associated with episodic verbal memory in patients with schizophrenia [153]. In patients with AD, delayed word recall test scores significantly correlated with rs11136000, one of the *CLU* gene SNPs [154]. Interestingly, the reduced episodic memory function that is associated with some CLU genotypes is attenuated by physical activity [155]. CLU protein levels increase in exercised mice, increasing memory performance and reducing brain inflammation [156]. 

## 4. Clusters of Cognitive Function-Associated Genes 

Cognition-related genes were downloaded from the DisGeNet website and used for PPI network analysis. In total, 308 genes were at the intersection of AD, T2DM, and memory function genes and were applied to the STRING database for a new round of analysis (Table S1). In total, 61 clusters were identified using the Markov clustering algorithm. Table S5 contains the list of the clusters and their proteins. As in Section 3, these clusters were sorted according to the average local clustering coefficient. 

### 4.1. Cluster 1

**Figure 2:**
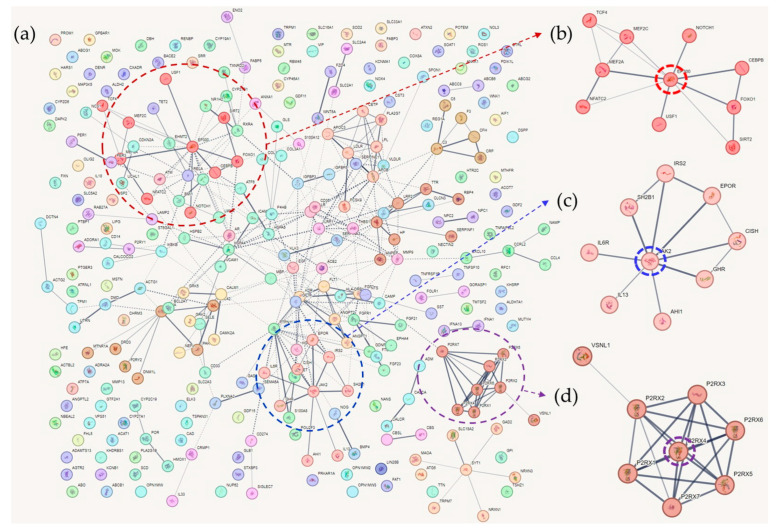
Network-based clustering of common genes of Alzheimer’s disease, type 2 diabetes, and cognition-associated genes. In (**a**), several clusters were distinctly detected. In Cluster 1 (**b**), Cluster 2 (**c**), and Cluster 3 (**d**), EP300, JAK2, and P2RX4 were hub proteins, respectively.

Cluster 1 contained 10 tightly interconnected genes (Figure 2). EP300 was the hub gene of the cluster. Mutational studies have shown that EP300 is associated with cognitive function. Mutations in EP300 have been reported in patients with Rubinstein–Taybi syndrome, which is characterized by cognitive impairment [157,158]. Fragile X syndrome protein (FMRP) is associated with EP300, and the loss of FMRP increases EP300 and HDAC1 levels in adult NSCs, resulting in age-related NSC depletion and cognitive impairment in mouse models [159]. EP300 expression is not activated when PS1 is mutated, and EP300 is involved in histone acetylation of PS1 and BACE1, which are key genes in AD pathogenesis [160]. It has been reported that EP300 and IL-17A are activated in SH-SY5Y cells and that inhibition of EP300 improves cognitive impairment [161]. Elevated EP300 activity is associated with an aberrant accumulation of immature autophagy markers, and blocking EP300 increases autophagy flux, reduces tau production, and decreases tau propagation [162]. In T2DM, overactivation of EP300 has also been identified; it is related to muscle atrophy by autophagy inhibition [163].

FOXO1 is a transcription factor involved in gluconeogenesis via insulin signaling [164]. Therefore, FOXO1 is closely linked to T2DM. Previous studies have reported that FOXO1 is involved in various mechanisms that cause beta cell dysfunction, including oxidative stress and cytokine induction [165,166]. Autophagy and FOXO1 are associated with beta cell viability, apoptosis, and insulin resistance [167]. Furthermore, FOXO1 is considered a potential therapeutic target for T2DM [168]. Reduced insulin receptor and insulin-like growth factor-1 receptor signaling decreased Aβ toxicity in a rodent model, which might have been induced by FOXOs, especially by FOXO1 and FOXO3 [169]. FOXO1 is involved in the autophagy of neurons [170], and the rs7981045 SNP variant of FOXO1 is associated with poor responses to acetylcholine esterase inhibitor treatment in patients with AD [171]. MiR-181a is a specific miRNA associated with cognitive function in pentylenetetrazol-induced epileptic rats [172], and miR-181a expression is reduced in APP^-^/PS1^-^ mice. MiR-181a has a protective effect against Aβ accumulation, but this effect is suppressed by FOXO1 [173]. When blood miRNA profiling was used to build a model for predicting the conversion from MCI to AD, FOXO1 was one of the four hub genes revealed by a network-based meta-analysis of microRNA expression quantitative trait loci target genes (involving expression variations) [174].

Notch1 is a transmembrane receptor that interacts with APP [175]. The proteolytic cleavage of Notch1 is affected by PS1 and Rac1 [176], and alterations to this process caused by gamma secretase may cause AD [177]. Furthermore, Notch1 affects neuronal progenitor cell differentiation [178]. It has been observed that elevated transcription of the intracellular domain of Notch1 restores the self-renewal activity of murine neuronal progenitor cells induced by PSEN1 mutations [178,179,180]. In addition, folic acid was shown to stimulate hippocampal neurogenesis in adult rat brains after ischemic injury [179], and Notch1 expression is reduced in the subventricular zone of ischemic aged brains of rats [180]. Downstream signaling of Notch1 is mediated by HES-1 and Hey-1, which bind to the insulin-degrading enzyme (IDE), a protein involved in the proteolytic cleavage of Aβ protein [181]. Moreover, IDE levels decreased when the intracellular domain of Notch was transfected into cell lines expressing human APP. In humans, immunohistochemistry identified Notch1 accumulation in the brain tissue of patients with sporadic AD [182]. Notch1 signaling is associated with cognitive function in AD [183], and several agents, including a hormone (melatonin) and a variety of chemicals (such as asiatic acid, risperidone, and valproic acid), affect cognitive function via Notch1 [184,185,186]. In diabetic rats and high glucose induced HepG2 cells, Notch1 is downregulated. When an inhibitor of miR-363 was applied to HepG2 cells, glucose consumption and uptake increased while lipid droplet accumulation decreased [187]. Additionally, salsalate is an anti-inflammatory drug with an antidiabetic effect, and its protective effect is diminished by the suppression of Notch1 [188].

### 4.2. Cluster 2

In cluster 2, JAK2 was an obvious hub gene linked to all the other genes in the cluster (Figure 2). JAK2 is associated with Aβ-induced hepatic insulin resistance. When Aβ is injected into the peritoneum of AD mouse models, it activates the hepatic Jak2/STAT3/SOCS-1 pathway, resulting in elevated fasting glucose and impaired insulin tolerance and hepatic insulin signaling [189]. When SH2B1 was knocked down, insulin expression and glucose-stimulated insulin levels decreased, and the reverse phenomena were observed with the overexpression of SH2B1 in rat beta cells [190]. Egr2 represses the expression of SOCS-1 and the phosphorylation of JAK2 and STAT3 in HepG2 cells following palmitate treatment, and Egr2 upregulation induces insulin resistance in HepG2 cells [191]. A high-fat diet is known to induce lipotoxicity in islet beta cells, which is associated with reduced PDX-1 expression, while the glucagon receptor agonist liraglutide induces the expression of PDX-1, JAK2, and STAT3, restoring insulin capacity and increasing the number of islet beta cells [192]. The antidiabetic effects of bromocriptine and the renoprotective effects of baricitinib, together with recombinant anti-IL-6 receptor proteins, were found to be associated with JAK2 inhibition [93,193,194]. IL-3 activates JAK2 and STAT3 in microglia, and this activation is associated with AD [195]. Inhibition of JAK2/STAT3 induced loss of spatial working memory through reduced levels of choline esterase and desensitization of the acetylcholine receptor [196]. Beta-amyloid downregulated IGF-1 expression by inhibiting the JAK2/STAT5 pathway in the adult rabbit hippocampus [197], and JAK2 inhibitors decreased PGE2 release and microglial phagocytosis [198]. When BDNF/TrkB activity is repressed, the JAK2/STAT3 axis activates, resulting in upregulation of C/EBPβ. This process is associated with increased δ-secretase and APP levels and tau fragmentation [199]. The JAK2/STAT3 cascade plays a crucial role in astrocyte reactivity, a hallmark of AD pathology [200]. 

IRS2 mediates the activation of the PI3K/Akt and MAPK pathways in insulin target tissues, and IRS2 knockout induces insulin resistance and beta cell degeneration [201]. Furthermore, IRS2 is involved in the autocrine regulation of insulin gene expression in beta cells [202]. In addition, beta cell survival is regulated by IRS2 expression and calcium ions [203], and the calmodulin-dependent kinase 4 (CaMKK)/CREB/IRS2 cascade stimulates beta cell survival in mice [204]. Calcineurin/NFAT signaling controls glucose-induced IRS2 expression in rat beta cells [205]. Notably, IRS2 mediates hepatic gluconeogenesis suppression through HIF2α- and VEGF-induced inhibition effects on glucose tolerance [206]. Prolyl hydroxylase domain-containing protein isoforms, including Phd1, Phd2, and Phd3, regulate the anabolic effect of insulin, and deletion of hepatic Phd3 improves insulin sensitivity by increasing *Irs2* transcription and *Akt* activation [207]. IRS2 is closely associated with amyloid pathology in AD. In mice with amyloid overexpression, deletion of *Irs2* reduced Aβ deposition by increasing clearance [208]. This finding was replicated in another study showing that the beneficial effect of Irs2 deletion was associated with IGF1 signaling alterations in AD mice [209]. Moreover, premature death of AD mice was prevented by *Irs2* deletion [209]. In contrast, decreased levels of IRS1 and IRS2 have been observed in the neurons of AD patients with aberrant IGF1R distributions [210]. Pathological changes in IGF1, IRS1, and IRS2 seemed to precede amyloid accumulation in an AD mouse model [211]. Recently, IRS2 was shown to play a predominant role in the brain insulin/IGF1 signaling pathway [212], and abscisic acid was found to affect hippocampal BDNF, TNFα, and IRS2, showing protective effects against AD [213].

IL-6R, which has a tight connection with JAK2, was a hub gene of Cluster 2. In the Chinese Han population, IL-6R gene polymorphisms have been associated with the onset of sporadic AD [214]. In contrast, Asp homozygotes of functional polymorphisms in IL-6R (Asp358Ala) were associated with higher cognitive performance [215]. Moreover, an IL-6R-responsive gene signature increased in the presence of IL-6R variant rs2228145, indicating the functional implications of IL-6R [216]. Additionally, the Asp358Ala variant of rs2228145 and elevated soluble IL-6R levels were associated with lower scores in modified preclinical Alzheimer’s cognitive composite and Montreal cognitive assessment [217]. When tocilizumab, an anti-IL-6R receptor, was administered to streptozotocin-induced AD mice, learning and spatial memory significantly improved [218]. In a human study, genetic variants of IL-6R were associated with the development of T2DM [219,220,221]. Inhibition of IL-6R by miR-22 augmented the viability of pancreatic cells and reduced the expression of apoptosis-related proteins [222]. 

### 4.3. Cluster 3

In Cluster 3, purinergic receptors were tightly connected (Figure 2). Purinergic receptors are involved in ATP-mediated signaling pathways [223]. There are three subtypes: P1, P2X, and P2Y. These receptors play different roles in a variety of biological processes, and Cluster 3 contains all types of P2RXs (P2RX1–P2RX7), which are ligand-gated ion channel receptors [223]. P2RX4 appears to be a hub gene of this cluster; however, few studies have reported an association between P2RX4 and AD or T2DM. Microglial P2XR4 regulates cathepsin B activity and promotes ApoE degradation, and deletion of P2XR4 recovers spatial memory impairment in mouse models [224]. OXYS rats, an advanced AD murine model, showed increased expression of *p2xr4* [225]. Aβ fragment 1-42-induced neuronal death in rodents is enhanced by upregulation of P2XR4 expression [226]. Not a single study reporting a relationship between P2XR4 and T2DM was found. 

Among the P2RXs, P2RX7 is the most frequently studied receptor. P2RX7 knockout mice show rapid postprandial hyperglycemia and increased beta cell apoptosis [227]. Additionally, the fibroblasts of patients with T2DM show increased expression of P2XR7 and accompanying cellular responses, such as enhanced fibronectin and IL-6 secretion, as well as activation of apoptosis [228]. The genetic variant rs1718119 of P2XR7 is associated with insulin sensitivity and secretion [229], increased beta cell function, and the release of IL-1Ra in patients with T2DM [230]. P2XR7 is associated with ATP-mediated pathophysiology of AD. In rats, when ATP is administered to primary microglia, P2XR7 mediates the stimulation of superoxide production, and microglia-induced cortical cell death occurs [231]. P2XR7 is also involved in the secretion of cytokines in microglia [232], and the activation of microglia by Aβ is accomplished by the upregulation of P2XR7, as observed in a transgenic mouse model of AD [233]. Furthermore, protein expression of P2XR7 in postmortem human brain samples has also been observed; it modulated the NLRP3 inflammasome pathway [234]. P2XR7 activation is associated with neuronal autophagy and cognitive and memory impairment after traumatic brain injury [235]. In tau transgenic mice, P2XR7 induces exosome secretion by microglia, and blockade of P2XR7 reversed cognitive deficits in the Y-maze, prepulse inhibition, and contextual fear conditioning tests [236].

*VSNL1* is located at the periphery of the P2 receptor network in this cluster; however, its role as a biomarker of AD is well known. Visinin-like protein 1 (VILIP-1) is encoded by the *VSNL1* gene; it acts as a neuronal calcium sensor protein and is involved in intracellular neuronal signaling [237]. VILIP-1 enhances tau protein hyperphosphorylation in P12 cells [238]. The *VSNL1* SNP variant rs4038131 is associated with psychotic symptoms in patients with AD, who are more prone to rapid cognitive decline [239]. VILIP-1 levels in the cerebrospinal fluid (CSF) have been shown to predict AD [240,241,242,243]. In addition, VILIP-1 levels predict the cognitive decline rates of patients with AD (measured by clinical dementia ratings and other scores) [243]. VILIP-1 levels in the CSF also help discriminate between patients with AD and patients with Lewy bodies, which are difficult to diagnose based on clinical symptoms [242], and these levels also have a predictive power for the differential diagnosis of AD and MCI, especially in conjunction with conventional biomarkers such as p-tau181 and Aβ(1-42) [241]. This finding was replicated in a meta-analysis of the association between VILIP-1 levels in CSF and AD [240]. While *VSNL1* and VILIP-1 have implications in the pathophysiology of AD, relatively few connections have been found between *VSNL1* and T2DM. VILIP-1 expression, for example, has an impact on the secretion of cyclic AMP (cAMP) and insulin in MIN6 cells and mouse islets [244]. Genetic fine mapping of quantitative expression traits using islet cell transcriptomics data revealed that *VSNL1* is a candidate T2DM risk gene [245]. However, no clinical studies have found an association between *VSNL1* and T2DM development, which should be investigated in future studies.

## 5. Discussion

The DisGeNet database is a collection of disease genes determined by experimental evidence, derived from molecular biology experiments and genetic association tests. The primary goal of the DisGeNet database is to provide information on disease genes that have already been validated. Therefore, we can identify genes associated with comorbidity directly through a simple intersection of disease genes from two diseases. However, the information from the database does not directly identify novel disease genes. Nevertheless, it can be valuable for predicting novel disease genes, especially through bioinformatic analysis. The primary goal of this review was to identify genes and clusters associated with AD, T2DM, and brain functions (memory and cognition) because genes involved in multiple diseases are more likely to be associated with diseases. By doing this, researchers can obtain more reliable targets for future research on AD biomarkers or drug development, specifically for addressing memory loss or cognition failure.

Since the number of disease genes associated with AD and T2DM is high, there is a possibility that the intersection of these two disease gene groups occurs randomly. To rule out this possibility, a statistical test based on random sampling was performed. Two sets of genes, each having the same number of AD and T2DM genes, were sampled from the pool of 43,161 human genes. The number of common genes was determined, and this process was repeated 100,000 times. No instance of a higher number of overlapped genes was observed for AD and T2DM by random chance (Supplementary Figure S1). This result indicates that the common genes between AD and T2DM cannot be obtained by random chance alone (permutation *p* value < 1 × 10^−5^). Additionally, over-representation analysis yielded highly significant results, indicating the biological significance of these genes in both AD and T2DM.

In this review, over-representation analysis with KEGG and GOBPs was applied to reveal the functional implications of common genes in AD and T2DM. Since these genes are associated with both diseases, they are highly likely to have functional implications. As anticipated, there were numerous highly significant results. The over-representation test assesses whether input genes have a greater number of genes in a gene set than would be randomly expected. The biological implication of significant enrichment is that input genes have functional genes associated with a specific biological process of the gene set. Although the statistical test uses the hypergeometric distribution, the significant results indicate that random sampling of the same number of input genes from the total amount of human genes does not yield more genes from the gene set. In this review, the enrichment test showed many highly significant results, indicating that the common genes in AD, T2DM, and memory (or cognition) have functional meaning, rather than being chosen by random chance.

The primary goal of this review was to identify clustered genes previously known to be associated with AD, T2DM, and memory/cognition based on the results of PPI network-based clustering. Considering the PPI network provides information on protein interactions determined by the physical properties of the proteins, genes in the same clusters are highly likely to be involved in the pathophysiology of related diseases. Given this, genes with relevant evidence guarantee functional relatedness to AD and T2DM, although the main focus can be on AD. Thus, in this review, listing the evidence related to the genes of the same cluster was the primary goal. The functional enrichment test of the STRING database supported this hypothesis. When the functional enrichment test was applied to the clusters from the STRING database, there were significant results that explained the functional characteristics of the cluster. This information can be applied to future research. For example, Cluster 1 (CL1) of common genes from AD, T2DM, and memory genes showed significant enrichment of the gene ontology biological process (GOBP) termed ‘Cardiac Ventricle Formation’ (false discovery rate < 0.05, Table S6). If a drug expresses activity on the genes of the GOBP term, it can be applied for repurposing research. Although additional information and considerations are necessary, the cluster information would be a useful starting point. Likewise, this review performed enumeration of relevant evidence of the cluster genes to ensure their usability, instead of summarizing individual molecular biologic evidence.

## 6. Conclusions

In this study, the genes related to AD and T2DM comorbidity were reviewed. Common comorbidity genes and genes affecting memory and cognition were used for PPI-based network clustering, and tightly connected gene clusters were thus obtained. Since common genes were detected with respect to different phenotypes, they were unlikely to be a randomly identified group. Moreover, instead of using comorbidity genes directly, the memory and cognition gene subset was used in analysis; therefore, the genes of the clusters are most likely involved in the pathophysiology of AD. Although the overall impact of the cluster genes on the entire genetic network of AD brain cells should be assessed for an accurate estimation of their roles in AD, these genes provide valuable guidelines for future research.

## Figures and Tables

**Table 1:** Over-representation analysis result with gene ontology.

GOBP ^1^	Odds Ratio	*p* Value
RESPONSE TO OXYGEN CONTAINING COMPOUND	16.06	1.97 × 10^−301^
POSITIVE REGULATION OF MULTICELLULAR ORGANISMAL PROCESS	13.616	1.24 × 10^−240^
RESPONSE TO ENDOGENOUS STIMULUS	12.81	2.40 × 10^−234^
CELLULAR RESPONSE TO OXYGEN CONTAINING COMPOUND	15.04	3.76 × 10^−224^
POSITIVE REGULATION OF SIGNALING	11.70	1.76 × 10^−222^
REGULATION OF TRANSPORT	11.53	3.81 × 10^−222^
REGULATION OF CELL DEATH	11.97	5.53 × 10^−222^
APOPTOTIC PROCESS	10.91	1.28 × 10^−219^
HOMEOSTATIC PROCESS	11.63	1.17 × 10^−216^
REGULATION OF CELL POPULATION PROLIFERATION	11.11	6.84 × 10^−212^

^1^ GOBP; Gene Ontology Biological Process.

**Table 2:** Over-representation analysis result with KEGG pathways.

KEGG ^1^ Pathway	Odds Ratio	*p* Value
PATHWAYS IN CANCER	21.62	9.22 × 10^−41^
NEUROTROPHIN SIGNALING PATHWAY	36.62	9.14 × 10^−32^
LEISHMANIA INFECTION	59.33	2.17 × 10^−30^
TOLL-LIKE RECEPTOR SIGNALING PATHWAY	41.26	1.18 × 10^−29^
CYTOKINE–CYTOKINE–RECEPTOR INTERACTION	18.45	1.46 × 10^−28^
FOCAL ADHESION	22.04	2.64 × 10^−27^
MAPK SIGNALING PATHWAY	17.61	3.42 × 10^−27^
COLORECTAL CANCER	59.67	1.10 × 10^−26^
APOPTOSIS	42.63	1.43 × 10^−26^
PROSTATE CANCER	41.36	2.49 × 10^−26^

^1^ KEGG; Kyoto Encyclopedia of Genes and Genomes.

## Data Availability

The data presented in this study are available in the Supplementary Materials.

## Supplementary Materials

The following supporting information can be downloaded at: https://www.mdpi.com/article/10.3390/ijms25042211/s1. References [246,247] are cited in the Supplementary Materials.
