# Supplementary material for: Comorbidity Genes of Alzheimer’s Disease and Type 2 Diabetes Associated with Memory and Cognitive Function
Source: Int J Mol Sci. 2024 Feb 12;25(4):2211. doi: 10.3390/ijms25042211 (PMC10889845; doi:10.3390/ijms25042211)

Supplementary Methods

Over-representation analysis with functional gene sets

Overrepresentation analysis (ORA) is a gene set analysis method that assesses whether specific genes within a gene set are enriched in a given list of genes. This method is primarily employed for the functional annotation of genes of interest. Fisher's exact test can be applied for statistical testing. ORA determines whether the genes of interest include more genes from functional gene sets, such as gene ontology or pathways, than expected by random chance.

	Genes of interest (+)	Genes of interest (–)	
Gene set (+)	*a*	*b*	*a*+*b*
Gene set (–)	*c*	*d*	*c*+*d*
	a+*c*	*b*+*d*	*a*+*b*+*c*+*d* (= *n*)

In the above table, the positive sign indicates that a gene is included in the list; the negative sign means the gene is not. n indicates the total number of human genes. Fisher's exact test identifies whether the distribution of numbers in the table results from random chance. The P-value of the test is obtained using the hypergeometric distribution, as shown in equation (1).

$$p = \frac{(a+b)!(c+d)!(a+c)!(b+d)!}{a!b!c!d!n!} \quad (1)$$

For the estimation of the p-value, the `'fisher.test'` function in the R program was used with the one-sided parameter.

Network-based clustering

In bioinformatics analysis, network-based clustering is employed to identify clusters with tightly connected genes or proteins, considering a specific network structure. In the STRING database, the Markov Cluster Algorithm (MCL) is implemented to detect clusters based on the protein-protein interaction network structure. MCL determines clusters by simulating a flow on the graph, with the

flow estimated through successive powers of the associated adjacency matrix. Strong and weak flow in the graph are contrasted by an inflation step in each iteration, ultimately partitioning the network into clusters. The default inflation parameter (set to 3) was used, and protein interactions were defined by physical interactions and confidence levels in the interactions.

Reference

Maleki,F. *et al.* (2020) Gene set analysis: challenges, opportunities, and future research. *Front. Genet.*, 11, 654.

Brohée S, van Helden J. Evaluation of clustering algorithms for protein-protein interaction networks. BMC Bioinformatics. 2006 Nov 6;7:488. doi: 10.1186/1471-2105-7-488. PMID: 17087821; PMCID: PMC1637120.

Supplementary Figure S1. Result of permutation analysis for number of common disease genes from Alzheimer's disease (AD) and type 2 diabetes (T2DM). Note that the red line indicates original number of common genes between AD and T2DM.

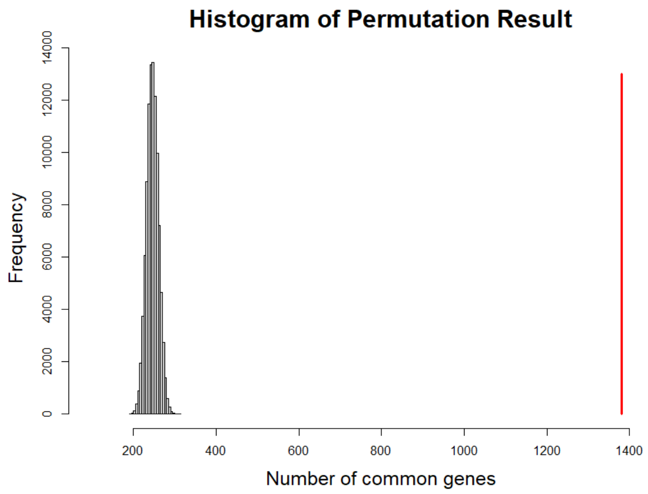

Supplement: Supplementary file 1 [file ijms-25-02211-s001.zip › Supplementary Methods.pdf]
